# The Impact of Diet, Nutrition and Nutraceuticals on Oral and Periodontal Health

**DOI:** 10.3390/nu12092724

**Published:** 2020-09-06

**Authors:** Gaetano Isola

**Affiliations:** Department of General Surgery and Surgical-Medical Specialties, School of Dentistry, University of Catania, Via S. Sofia 78, 95124 Catania, Italy; gaetano.isola@unict.it; Tel.: +39-095-3782453

**Keywords:** periodontitis, oral diseases, diet, nutrients, nutraceutics, therapy, host response

## Abstract

Oral and periodontal diseases can determine severe functional, phonatory and aesthetic impairments and are the main cause of adult tooth loss. They are caused by some specific bacteria that provoke an intense local inflammatory response and affect—with particular gravity—susceptible subjects, because of reasons related to genetics and lifestyles (e.g., smoking and home oral hygiene habits). They are more frequent in the disadvantaged segments of society and, in particular, in subjects who have difficulty accessing preventive services and dental care. Some systemic diseases, such as uncontrolled diabetes, can increase their risk of development and progression. Recently, in addition to the obvious considerations of severe alterations and impairments for oral health and well-being, it has been noted that periodontitis can cause changes in the whole organism. Numerous clinical and experimental studies have highlighted the presence of a strong association between periodontitis and some systemic diseases, in particular, cardiovascular diseases, diabetes, lung diseases and complications of pregnancy. The purpose of this editorial is to provide a current and thoughtful perspective on the relationship of diet and natural agents on oral, periodontal diseases, and chewing disorder preventions which may reflect good systemic conditions and related quality of life or to analyze indirect effects through the contribution of diet and nutrition to systemic health in order to obtain a modern diagnostic–therapeutic approach.

## Editorial

Periodontitis is a multifactorial disease in which both environmental and genetic factors play a precise and controversial role in determining its onset [1]. Oral bacterial flora certainly plays an important role in the progression of this pathology. Further risk factors, widely studied, are smoking and diabetes [2,3,4]. However, a series of genetic factors of the host can condition the individual susceptibility to the onset of the disease, determine its different clinical manifestations and the rate of progression [5,6].

Unlike Mendelian genetic diseases, which are rare and caused by a single or few mutations, multifactorial diseases, such as periodontitis, are frequent and related to numerous environmental and genetic factors. Genetic factors are not real mutations, but genetic polymorphisms, also called susceptibility factors. Each of these is not necessary or sufficient to determine the disease, however, they are able to modify the risk of its onset [7,8].

These polymorphisms are variations in the genetic code that can have different effects, for example, changing the levels of gene expression, causing slight functional changes of the coded molecules, making individuals more susceptible to the onset of a certain disease or to the appearance of clinical pictures more serious than the disease itself [9].

In recent years, investigations into susceptibility factors for the development of periodontal diseases have mainly focused on the study of genes that encode factors involved in modulating the immune response, cell surface receptors, chemokines, enzymes and proteins related to antigen recognition. Cytokines, such as IL-1A, IL-1B, IL-10 and IL-6, are key factors that mediate the inflammatory process in periodontal disease. They play a role in the activation, proliferation and differentiation of B cells, the main cells implicated in severe manifestations of periodontitis [10,11,12,13].

These genetic variations can, therefore, favour the progression of the disease, causing the classic trend, characterized by repeated cycles of tissue inflammation, followed by spontaneous remissions (defined as “pousses” trend) [14,15].

In periodontal disease, pathogenic bacteria accumulated in the subgingival sulcus are the environmental factors that influence the inflammatory response of the periodontal tissues [16,17]. However, a central role of diet, natural agents and nutraceutics are also considered indirectly responsible for the health of periodontal tissues and against alveolar bone resorption [18,19,20,21,22].

In this regard, Liu et al. [23] found that seven bacterial taxa, including Streptococcus sp., Ruminococcaceae sp., Haemophilus sp., Veillonella spp., Actinomyces odontolyticus, and Gemella haemolysans, were significantly altered after oolong tea consumption, and presented robust strong connections with other oral microbiota. These results suggest that sustained oolong tea consumption would modulate salivary microbiota and generate potential oral pathogen preventative benefits.

Since alveolar bone resorption is a key factor in periodontal disease, the vitamin D receptor (VDR) has been considered as a susceptibility factor in disease progression. Data in the literature support the existence of an association between common polymorphisms affecting candidate genes and periodontal disease [22,24,25].

Interestingly, most genetic studies of periodontitis have employed small cohorts. The limited statistical power of studies conducted with a low number of samples leads to an imprecise assessment of the level of genetic risk and the danger of obtaining false-positive and false-negative results.

Periodontitis develops severely in genetically predisposed individuals [26,27]. Genetic susceptibility is believed to be due to changes in the subject’s genes that lead to i) a lower efficiency of the immune system in controlling the growth of pathogenic bacteria; and/or ii) an imperfect regulation of the inflammatory response [28,29,30,31,32] which leads to an increase in the destructive side effects of inflammation [33,34,35,36]. As a matter of fact, Jekabsone et al. [37] explored antibacterial, antinflammatory and cytoprotective capacity of Pelargonium sidoides DC root extract (PSRE) and proanthocyanidin fraction from PSRE (PACN) under conditions characteristic for periodontal disease. They found that PSRE and especially PACN possess strong antibacterial, anti-inflammatory and gingival tissue protecting properties under periodontitis-mimicking conditions and are suggestable candidates for the treatment of periodontal disease.

Great importance is also attached to lifestyles [38]; first of all, smoking and home oral hygiene habits, orthodontic treatment [39,40,41,42] and malocclusions [43,44,45,46,47,48], as they explain at an epidemiological level a large portion of the cases of periodontitis and dental malocclusions [49,50,51] observed and are modifiable and therefore important for prevention and treatment.

The general state of health of the subject is another element that can increase the risk of developing periodontitis. For example, people with poorly controlled diabetes have three times higher risk than non-diabetics of developing periodontitis [52,53].

Bodgdan et al. [54] conducted a systematic review of clinical trials that measured plasmatic/salivary levels of ascorbic acid in PD–diabetes mellitus (DM) association. They found that decreased levels of vitamin C were observed in PD patients with DM but data about the efficacy of vitamin C administration are inconclusive. Given the important bidirectional relationship between PD and DM, there is a strong need for more research to assess the positive effects of ascorbic acid supplementation in individuals suffering from both diseases and also its proper regimen for these patients.

Moreover, in this aspect, Nastri et al. [55], in their scoping review, summarized the role of dietary supplements in optimizing osseointegration after implant insertion surgery. The authors concluded highlighting the limited role of nutraceuticals in promoting the osseointegration of dental implants. However, in some cases, such as for vitamin D deficiency, there is a clear link among their deficit, reduced osseointegration, and early implant failure, thus requiring an adequate supplementation.

Knowing the patient’s genetic profile or their predisposition to the disease could be very useful in diagnosing periodontal disease and in defining a personalized therapeutic plan. In addition, it could give prognostic indications of the outcome of the disease.

Data derived from epidemiological observations are therefore important to establish the existence of a relevant and stable association but are insufficient to demonstrate the causal link and therefore the general health benefits deriving from the treatment and prevention of periodontitis. Causality can only be demonstrated unequivocally in randomized controlled trials that include eliminating or reducing (through prevention or therapy) the exposure of subjects to the harmful effects of periodontitis: pathogenic bacteria and gingival inflammation. These studies must conform to the highest quality standards and test the therapy capable of reducing the exposure in a clinically relevant way for each systemic pathology for which a significant association has emerged.
